# Iodine Deficiency of Breastfeeding Mothers and Infants from 2012 to 2019 in Zhengzhou, China

**DOI:** 10.1007/s12011-022-03531-w

**Published:** 2022-12-20

**Authors:** Xinyi Wang, Jianghua Liu, Weixia Lu, Weihua Jia, Qingzhi Li, Stanislav S. Traoré, Quanjun Lyu

**Affiliations:** 1 Department of Nutrition and Food Hygiene, College of Public Health, Zhengzhou University, Zhengzhou, 450001 China; 2 Zhengzhou Center for Disease Control and Prevention, Zhengzhou, 450006 China; 3 Department of Nutrition, the First Affiliated Hospital of Zhengzhou University, Zhengzhou, 450052 China

**Keywords:** New iodine standard, Iodine deficiency, Urinary iodine concentration, Infants, Breastfeeding women

## Abstract

To investigate iodine status and characteristics of breastfeeding women and infants in Zhengzhou after the implementation of the new national standard of iodine in edible salt, so as to provide the basis for formulating prevention and control measures. Urine samples from 28,730 infants aged 0–2 years and 17,977 breastfeeding women who received preventive health care in 12 districts/cities of Zhengzhou from 2012 to 2019 were collected to measure urinary iodine concentration (UIC). A total of 350 pairs of unweaned infants and their mothers were included in this study. After the implementation of the new national standard of iodine in edible salt, the iodine deficiency of infants aged 0–2 years showed a trend of decreasing first and then increasing, but generally the iodine nutrition of infants aged 0–2 years was at the appropriate level in 8 years. There was a gradual decrease in iodine deficiency among breastfeeding women over an 8-year period. And the median UIC of breastfeeding women in 8 years was at iodine nutrition appropriate level. In addition, the UIC of breastfeeding mothers was positively associated with that of infants (*r* = 0.104, *P* = 0.004). After the implementation of the new national standard of iodine in edible salt, breastfeeding women and infants in Zhengzhou generally were at an appropriate level of iodine nutrition, and there was a significant positive correlation between the UIC of breastfeeding mothers and infants.

## Introduction

Iodine is an essential trace element for human beings and one of the key components for the synthesis of thyroid hormone [1]. The thyroid hormone regulates the metabolic process of most cells and plays an important role in human growth, development, and metabolism [2]. Chronic iodine deficiency can cause abnormal physical and mental development in infants and children, thus leading to cretinism [3]. Salt iodization has been recognized as an effective, convenient, and economical method of iodine supplementation [4], and is recognized as an important pillar of the global plan to eradicate deficiency diseases [5].

China used to be the largest country with iodine deficiency [6], and twelve districts and counties in Zhengzhou, Henan Province, are also iodine deficiency areas [7]. China began implementing the policy of “Universal Salt Iodization (USI)” in 1995 [8]. In the two decades since iodized salt was made mandatory, significant progress has been made and iodine deficiency has been greatly improved [9]. According to the National Survey on Iodine Deficiency Disorders (IDD), China has been on a sustainable path to eliminating IDD since 2005 [10]. As a result, interventions to combat IDD have also been geared towards preventing and controlling excessive iodine intake in the population. A community-based epidemiological survey of schoolchildren in China showed that the median urinary iodine was close to or higher than 200 μg/L in 2009–2010 [11]. Zhengzhou carried out iodine nutrition monitoring for key populations annually. According to the national iodine deficiency disease monitoring results in 2011 [12], the median urinary iodine concentration of children aged 8–10 years in Henan province was 201 μg/L. These data indicated that the iodine level of the general population in Henan province exceeded the suitable iodine range of 100 ~ 199 μg/L. Therefore, Since March 2012, Henan Province has implemented the national food safety standard “Iodine Content in Edible Salt,” and the iodized salt concentration has been reduced from the original (35 ± 15) mg/kg to 30 mg/kg ± 30% [13].

According to the survey, under the background of the universal implementation of salt iodization in China, there are still differences in iodine intake in different regions [14]. In addition, differences in iodine intake due to geographical distribution, dietary habits, and water iodine content are also of concern. According to the results of iodine nutrition monitoring of key population in 2019, the median UIC of children aged 8–10 years in Kunshan City, Jiangsu Province, Ningxia Hui Autonomous Region, and Beijing was 228.0 μg/L, 205 μg/L, and 173 μg/L, respectively [15–17]. Therefore, individualized assessment of the iodine deficiency of breastfeeding mothers and infants in Zhengzhou after the implementation of the new national salt iodine standard is very necessary for the follow-up adjustment of iodized salt in Henan Province.

Zero to 2 years old is also an important period of neural development for infants and young children. Adequate iodine intake is needed to maintain normal thyroid function and ensure brain development. Cao et al. [18] demonstrated that the intelligence quotient (IQ) of children in areas with severe iodine deficiency was 12.45 points lower than that of those in areas with sufficient iodine. A relevant meta-analysis also confirmed that the IQ scores of iodine-deficient children under 5 years old were 6.9 to 10.2 points lower than those of iodine-adequate children [19]. The World Health Organization recommends exclusive breastfeeding within 6 months after birth [20]. Iodine nutrition for breastfed infants depends on the amount of iodine in breast milk to ensure optimal thyroid function during the first 6 months of life [21]. In an experimental study of the effects of iodine deficiency on the brain development of neonatal rats, it was found that severe iodine deficiency in female rats significantly reduced the mRNA and protein levels of protein-2 in Purkinesia cells of neonatal rats, suggesting cerebellar retardation [22]. In a meta-analysis, the results showed that iodine supplementation during pregnancy can improve the iodine status in pregnant women and their offspring [23]. Therefore, iodine deficiency in breastfeeding mothers will affect the iodine nutrition of infants to a certain extent [24].

Therefore, we conducted this study to evaluate the iodine deficiency status of 0 ~ 2-year-old infants and breastfeeding women from 2012 to 2019, and to investigate the effect of maternal iodine nutritional status on their offspring.

## Methods

### Participants

This is a cross-sectional study. In each county (city, district) of Zhengzhou, one hospital, a center for disease control and prevention, and a community service center carrying out vaccination services were randomly selected. The participants included in this study were 0–2-year-old infants who were vaccinated and their mothers. The number of breastfeeding women and infants aged 0 to 2 years was surveyed each year. The inclusion criteria for the participants in this study were (1) healthy cases and (2) residents in each district or county of Zhengzhou for at least 6 months. Exclusion criteria were as follows: (1) multiple births and (2) a history of long-term medical conditions and medication. Because complementary foods for infants older than 6 months of age may affect their iodine intake, only exclusively breastfeeding mothers who had not consumed iodine-rich foods in the last 3 days and their infants (*n* = 350) up to 6 months of age were matched for analysis.

The Medical Ethics Committee of Zhengzhou Center for Disease Control and Prevention approved the study and was in accordance with the Declaration of Helsinki. Written informed consent was obtained from all mothers.

### Collection of Questionnaires

The face-to-face structured questionnaire completed by trained researchers was adopted to collect the following information: (1) sociodemographic information: mother’s age, occupation, family monthly income, and infant feeding methods; (2) the UIC of infants and their mothers; (3) mother’s intake of iodine-rich food in recent 3 days: iodine-rich food is considered to be seaweed, kelp, and other seafood.

### Laboratory Analysis

Approximately 10–20 mL mid-stream urine was collected from each subject in the morning, which was sealed in sterile polyethylene plastic test tubes and stored in an icebox at 4 to 8℃, and the collection date and time were recorded. UIC was tested using “WS/T 107–2006 Arsenic-Cerium Catalytic Spectrophotometric Determination of Iodine in Urine” [25]. The external quality control was provided by the China National Clinical Iodine Deficiency Disorders Reference Laboratory. The results showed that the coefficient of variation of UIC in our laboratory was 2.0% at 68.2 ± 1.3 μg/L and 0.9% at 193.0 ± 10.0 μg/L.

### Definition of Iodine Deficiency

According to the evaluation standard of iodine nutritional status of the population recommended by WHO, UNICEF, and ICCIDD [26], a median urinary iodine level < 100 μg/L in breastfeeding women and infants aged < 2 years indicate iodine deficiency.

### Statistical Analysis

The SPSS (Version 21.0.0.0, IBM, America) was subsequently used for statistical description and statistical analysis of the data obtained. UIC was not normally distributed; thus, the results were described for the median (25th percentile, 75th percentile). Numbers (percentages) for the categorical variables. The chi-square test was used to compare iodine deficiency rates among different groups. The Kruskal–Wallis test was used to compare urinary iodine between multiple groups. If the difference was statistically significant, pairwise comparisons were conducted and Bonferroni was used to correct the* P* values. Spearman was used for correlation analysis. Prism (Version 9.0.0, GraphPad Software, LLC, America) was used to plot the trend. Two-sided *P* values < 0.05 were considered statistically significant.

### Quality Control

Before the investigation, the on-site investigators were trained, the investigation methods and implementation rules were uniformly stipulated, and special personnel were programmed to be responsible for sample collection, laboratory testing, and laboratory quality control. Laboratories handling urine iodine testing were required to pass an external quality control assessment program before they could carry out laboratory testing. In the process of testing, the standard curve and quality control samples were to meet relevant requirements.

## Results

### General Characteristics of Infants and Breastfeeding Women

**Fig. 1:**
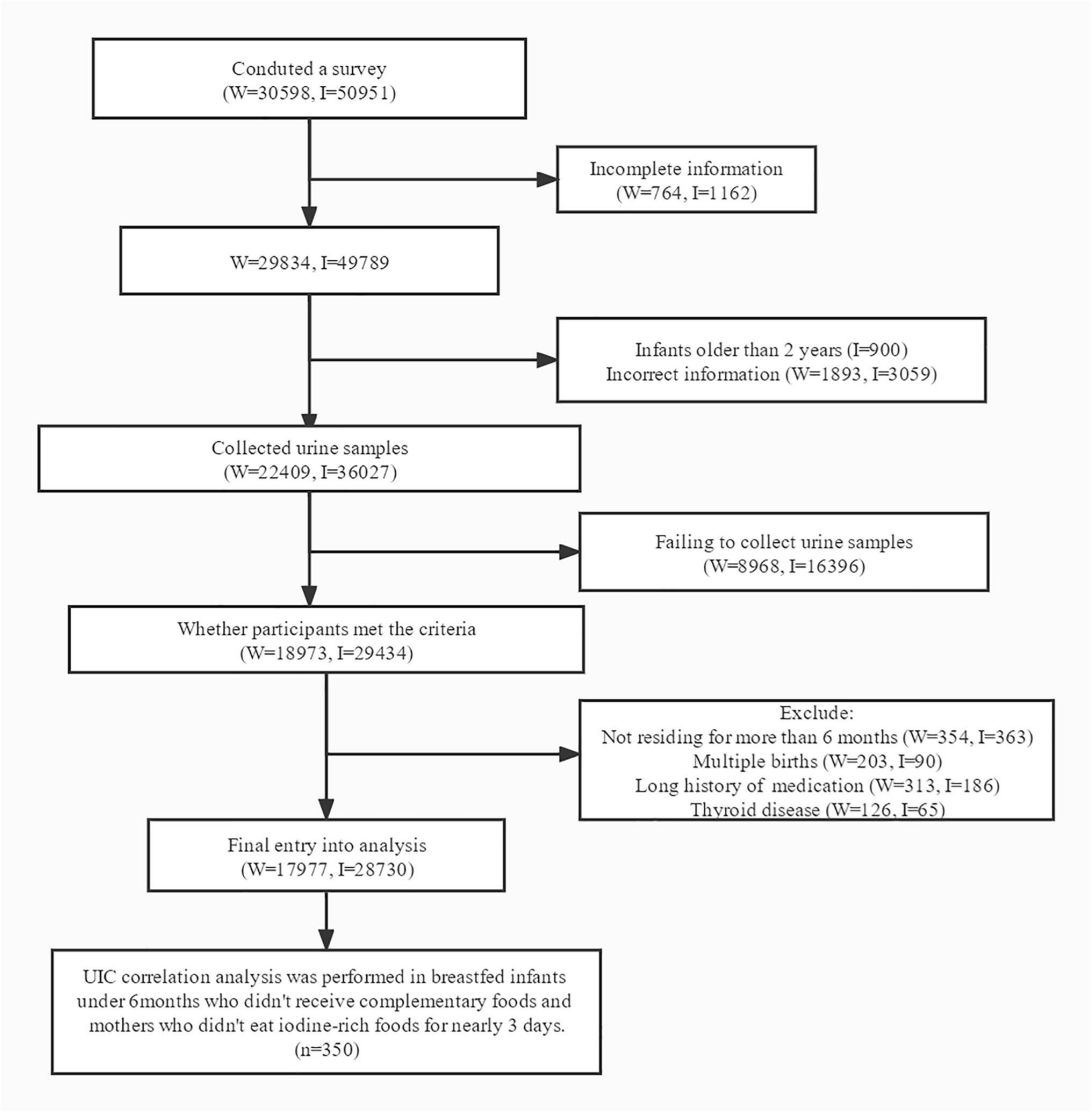
The flow chart of the inclusion process. *W* means breastfeeding women; *I* indicates infants

Maternal and infant characteristics are shown in Table 1. A total of 28,730 0–2-year-old infants (57.5% boys) were recruited from 2012 to 2019, of whom 44.2% were breastfed. Correspondingly, a total of 17,977 breastfeeding mothers were included, with an average age of 27.25 ± 4.7 years (range: 16–47 years). The flow chart of the inclusion process is shown in Fig. 1.

**Table 1:** Characteristics of infants and breastfeeding women

Maternal and infant variables	Values
Breastfeeding women (*n* = 17,977)	
Age (years)	
< 20 (%)	1.3
20 ~ (%)	72.9
30 ~ (%)	23.9
40 ~ (%)	1.9
Residential type	
Urban (%)	35.1
Rural (%)	64.9
Year (*n*)	
2012	4389
2013	3978
2014	3455
2015	1493
2016	1239
2017	1136
2018	1125
2019	1162
Infants (*n* = 28,730)	
Boy (%)	57.5
Age (months)	
0 ~ 3 (%)	61.1
4 ~ 6 (%)	15.0
7 ~ 12 (%)	14.4
13 ~ 24 (%)	9.5
Residential type	
Urban (%)	41.9
Rural (%)	58.1
Feeding pattern	
Breastfeeding (%)	44.2
Artificial feeding (%)	29.4
Mixed feeding (%)	26.4
Year (*n*)	
2012	3008
2013	1916
2014	6978
2015	6405
2016	3655
2017	4363
2018	1416
2019	989

### Iodine Status Among Infants and Associations with Their Characteristics

A total of 28,730 urine samples of infants aged 0 to 2 years were tested, among which the iodine deficiency rate was 4.9% in boys and 5.7 in girls. The sex distribution difference was statistically significant (*P* = 0.001). In terms of age range, the iodine deficiency rate was 3.4% for infants aged 0 to 3 months, 3.9% for infants aged 4 to 6 months, 7.6% for infants aged 7 to 12 months, and 15.7% for infants aged 13 to 24 months. The age distribution of iodine deficiency was statistically significant between 0 to 3 months, 7 to 12 months, and 13 to 24 months (*P* < 0.05), and there was no significant difference between 4 and 6 months of age (*P* < 0.05). The distribution of iodine deficiency in infants aged 0 to 2 was different between urban and rural areas (*P* = 0.001). The details are shown in Table 2.

**Table 2:** Iodine deficiency among infants

	The rate of iodine deficiency (%)	*χ*^2^	*P* value
Gender			
Boy	4.9	10.474	0.001
Girl	5.7		
Age (months)			
0 ~ 3^a^	3.4	779.755	< 0.0001
4 ~ 6^a^	3.9		
7 ~ 12^b^	7.6		
13 ~ 24^c^	15.7		
Residential type			
Urban	5.8	10.878	0.001
Rural	4.9		

There was no statistically significant difference between the two groups with the same superscript letters a, b, and c, but there was a difference between the two groups with different superscript letters

The iodine deficiency rate of infants with different feeding methods in different age groups was compared (Table 3), and the difference in iodine deficiency rate in infants with different feeding methods was statistically significant from 0 to 3 months (*P* = 0.001). The iodine deficiency rate of artificially fed infants was significantly higher than that of the other two groups. The iodine deficiency rate of infants with different feeding methods in different age groups was compared, and the difference in iodine deficiency rate in infants with different feeding methods was statistically significant from 0 to 3 months. The iodine deficiency rate of artificially fed infants was significantly higher than that of the other two groups (*P* > 0.05).

**Table 3:** Iodine deficiency in infants of different feeding styles at different ages

Age (months)	Feeding types	Number	The rate of iodine deficiency (%)	*χ*^2^	*P* value
0 ~ 3	Breastfeeding^a^	8556	3.3	13.35	0.001
	Artificial feeding^b^	4211	4.2		
	Mixed feeding^a^	4780	2.8		
4 ~ 6	Breastfeeding	1785	3.7	3.110	0.211
	Artificial feeding	1565	4.5		
	Mixed feeding	951	3.2		
7 ~ 12	Breastfeeding	1527	6.7	2.751	0.253
	Artificial feeding	1572	7.9		
	Mixed feeding	1034	8.4		
13 ~ 24	Breastfeeding	828	12.6	5.395	0.067
	Artificial feeding	1102	15.9		
	Mixed feeding	819	13.0		

There was no statistically significant difference between the two groups with the same superscript letters a and b, but there was a difference between the two groups with different superscript letters

### Trend in UIC Across Time Among Infants

**Fig. 2:**
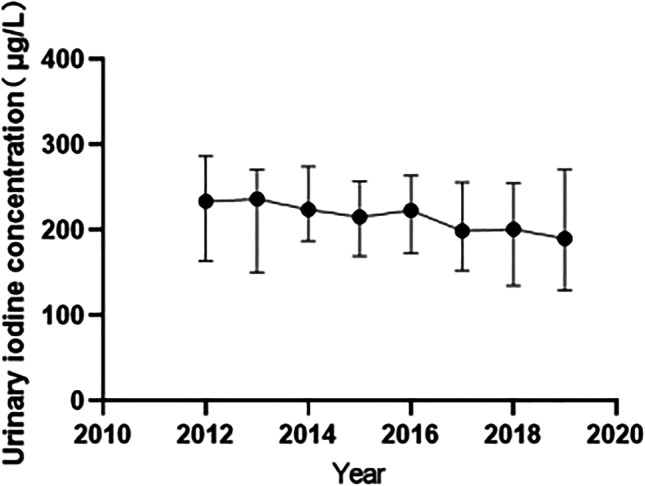
Trend of the UIC of infants aged 0–2 years in Zhengzhou from 2012 to 2019

The mean or appropriate level of UIC in infants aged 0–2 years was 217.65 μg/L. From 2012 to 2019, there were differences in the number of infants aged 0–2 years with iodine deficiency in Zhengzhou during the 8 years (*P* < 0.001) (Table 4). From the line chart, the median UIC of infants aged 0–2 years was relatively stable and at the appropriate iodine level (Fig. 2), indicating that infants aged 0–2 years in Zhengzhou had a good iodine nutritional status.

**Table 4:** Trend in UIC among infants aged 0–2 years from 2012 to 2019

Year	Number	UIC (μg/L)		*H*	*P* value
		Median	*P*_25_ ~ *P*_75_		
2012	3008	233.79	163.41 ~ 286.76	536.075	< 0.0001
2013	1916	236.39	150.48 ~ 270.79		
2014	6978	224.27	187.52 ~ 274.57		
2015	6405	215.59	169.46 ~ 257.62		
2016	3655	223.08	172.77 ~ 263.94		
2017	4363	199.23	152.79 ~ 256.04		
2018	1416	201.00	135.21 ~ 254.80		
2019	989	190.00	129.00 ~ 271.05		
Total	28,730	217.65	166.51 ~ 267.62

### Iodine Status of Breastfeeding Mothers and Associations with Their Characteristics

A total of 17,977 urine samples from breastfeeding women were tested, with an iodine deficiency rate of 16.4%. The iodine deficiency rates were 17.5%, 16.1%, 17.3%, and 16.4% for those under 20 years of age, 20–29 years of age, 30–39 years of age, and over 40 years of age. There was no difference in age distribution among breastfeeding women with iodine deficiency (*P* = 0.259). There was no significant difference in the distribution of iodine deficiency in breastfeeding women between urban and rural areas (*P* = 0.237). The details are shown in Table 5.

**Table 5:** Iodine deficiency among breastfeeding women

	The rate of iodine deficiency (%)	*χ*^2^	*P* value
Age (years)			
< 20	17.5	4.019	0.259
20 ~	16.1		
30 ~	17.3		
40 ~	16.4		
Residential type			
Urban	16.8	1.406	0.237
Rural	16.2		

### Trend in UIC Across Time Among Breastfeeding Women

**Fig. 3:**
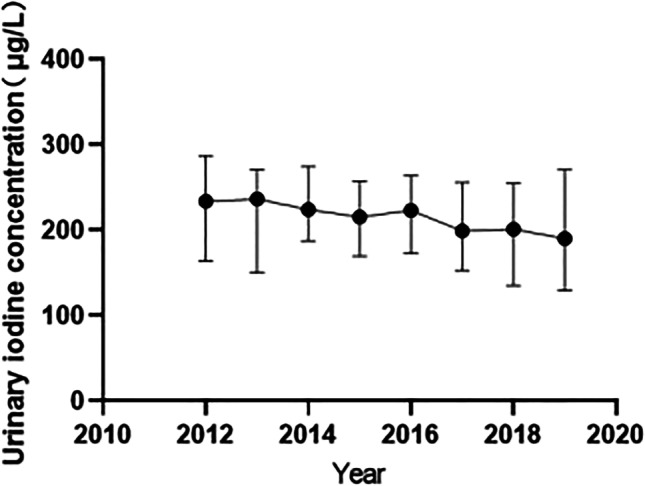
Trend of the UIC of breastfeeding women in Zhengzhou from 2012 to 2019

From 2012 to 2019, there were significant differences in the number of breastfeeding women with iodine deficiency in Zhengzhou during the 8 years (*P* < 0.001) (Table 6). From the line chart, the median urinary iodine of breastfeeding women was at a relatively stable level and at the appropriate iodine level (Fig. 3), indicating that the iodine nutritional status of breastfeeding women in Zhengzhou was good.

**Table 6:** Trend in UIC among breastfeeding women from 2012 to 2019

Year	Number	UIC (μg/L)		*H*	*P* value
		Median	*P*_25_ ~ *P*_75_		
2012	4389	160.94	104.82 ~ 253.51	50.251	< 0.0001
2013	3978	163.13	110.25 ~ 237.53		
2014	3455	167.88	134.15 ~ 226.30		
2015	1493	164.37	125.66 ~ 219.97		
2016	1239	166.81	113.78 ~ 233.10		
2017	1136	155.18	119.99 ~ 206.48		
2018	1125	173.07	116.10 ~ 236.28		
2019	1162	168.30	117.00 ~ 234.01		
Total	17,977	164.57	117.09 ~ 233.03

### Concordance Between Iodine Status Among Infants and Their Mothers

**Fig. 4:**
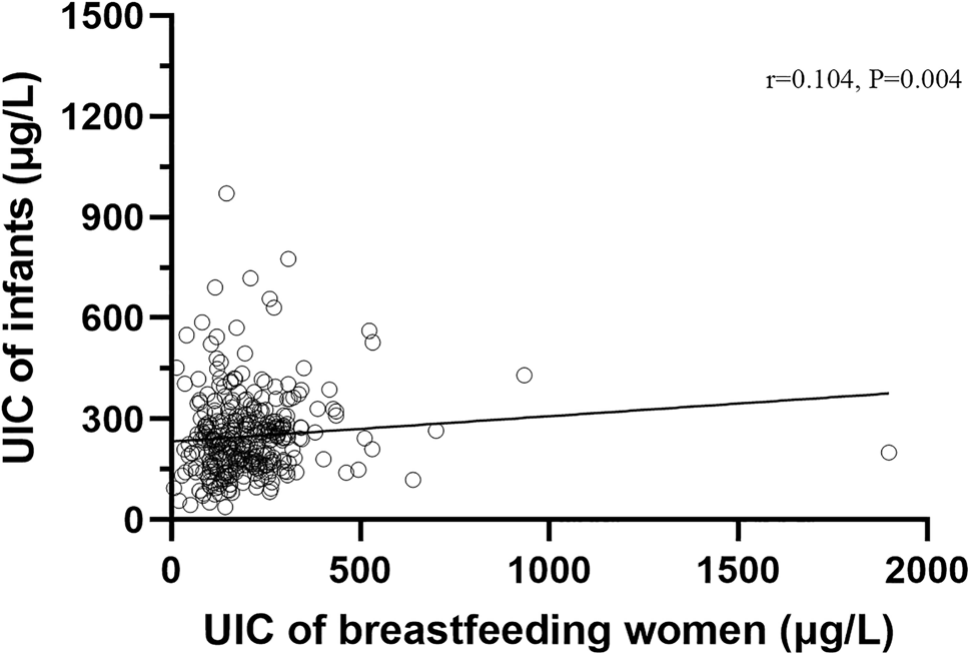
Correlation analysis between the UIC of 0 ~ 2-year-old infants and their breastfeeding mothers

Of all the participants, 350 pairs of unweaned infants and their mothers were matched. As is shown in Fig. 4, paired correlation analysis was conducted to compare the UIC between breastfed infants who had not been weaned and their breastfeeding mothers. The results showed that there was a significant positive correlation between breastfed infants and their mothers (*n* = 350, *r* = 0.104, *P* = 0.004).

## Discussion

Iodine is one of the essential trace elements in the human body and plays an important role in human growth, development, and metabolism [27]. The definition of iodine nutrition status in the population recommended by WHO is an important indicator to evaluate the iodine nutrition level of the population, which can reflect the recent iodine nutrition status [28]. Iodine deficiency has resurfaced in some developed countries, especially in pregnant and breastfeeding women and children [29]. Through the implementation of this study, we found that the iodine nutrition level of lactating women and infants in Zhengzhou, China, is generally at an appropriate level. When a breastfeeding woman is iodine-deficient, the amount of iodine available to the baby through breast milk decreases, resulting in the baby’s own iodine deficiency, which affects the development of the nervous system. In this study, it is found that the UIC of breastfeeding mothers and infants is significantly positively correlated (*r* = 0.104, *P* = 0.004). In a randomized controlled trial conducted in Ningxia Hui Autonomous Prefecture [30], infants weaned under 3 years of age in the intervention group were given 40 mg iodized oil every year for 4 consecutive years. It was found that the developmental quotient (DQ) value increased from 92.8 to 104.3, the proportion of normal height and above increased from 65.0 to 82.1%, and the proportion of normal weight and above increased from 59.3 to 81.4%. DQ value, height, and weight were significantly different from those before the intervention (*P* < 0.05). Therefore, appropriate iodine levels are critical for breastfeeding women and infants.

According to the urine iodine standard recommended by WHO, the urine iodine status of key populations in Zhengzhou City during the past 8 years was evaluated and the iodine nutrition of breastfeeding women and infants was found to be in an appropriate state. Monitoring of infants found that there was a statistically significant difference in urinary iodine in infants of different ages, and the number of people with iodine deficiency increased with age, which was consistent with the findings of Song et al. [31], because the potential reason might be that infants get less iodine from breast milk as they get older [32], and some parents believe that salt should be avoided in supplement food for infants [33]. Results of this study on the effects of different feeding methods at different ages on infant iodine deficiency showed that the iodine deficiency rate of infants with mixed feeding at 0–3 months was significantly higher than that of infants with breastfeeding and mixed feeding, while there was no difference in the distribution of iodine deficiency in other ages. This is consistent with other research finding that formula-fed infants are more likely to develop iodine deficiency than breastfed infants [34, 35]. In addition, the protective effect of breastfeeding against iodine deficiency in infants aged 0–3 months is more pronounced, possibly because of the gradual introduction of complementary foods at 4–6 months.

Since March 2012, Henan Province has implemented the national food safety standard “Iodine content in Edible Salt,” and the iodized salt concentration has been adjusted from the original (35 ± 15) mg/kg to 30 mg/kg ± 30% [13]. According to the survey results from 2012 to 2019, the median urine iodine of breastfeeding women and infants aged 0–2 years in Zhengzhou has reached the appropriate iodine level every year, maintaining the elimination of iodine deficiency disease. In this study, the correlation analysis of urine iodine concentration of breastfed infants and their mothers showed that there was a significant positive correlation between the urine iodine concentration of breastfeeding mothers and infants, which was consistent with the results of some studies [1, 24, 36, 37]. The results showed that the iodine nutritional status of breastfeeding mothers could reflect the iodine nutritional status of infants.

However, there are still some limitations in this study. Firstly, the lack of 24 h urine iodine data from key participants involved in the study. Iodine nutritional status is considered to be most closely estimated by the amount of iodine excreted in urine over 24 h [38]. Secondly, due to the lack of iodine deficiency data of breastfeeding women and infants aged 0–2 years in Zhengzhou before 2012, the trend of UIC after the implementation of the new national salt iodine standard was not compared. In addition, we did not investigate the use of iodine supplements in lactating women and could not rule out the effect of iodine supplements on UIC. In future studies, we need to use a generalized linear logistic regression model to further explore the multivariate effects of dietary habits of iodine intake, the use of iodine supplements, and geographical location on iodine deficiency.

In conclusion, our study shows that after the implementation of the new national standard of iodine in edible salt, the iodine deficiency of breastfeeding women in Zhengzhou is gradually reduced, while the iodine deficiency of infants still needs to be improved by measures, and there is a significant positive correlation between the UIC of breastfeeding mothers and infants. In the future, in addition to the continuous implementation of the national salt iodization strategy, more efforts are urgently needed, such as strengthening iodine nutrition monitoring among key populations and paying attention to behavioral intervention with iodine supplementation among key populations, so as to improve the iodine deficiency situation among them.

## Data Availability

For raw data for this study, please contact the corresponding author.
